# Vibrational Properties of Nanocrystals from the Debye Scattering Equation

**DOI:** 10.1038/srep22221

**Published:** 2016-02-26

**Authors:** P. Scardi, L. Gelisio

**Affiliations:** 1 University of Trento, Department of Civil, Environmental and Mechanical Engineering, Trento, 38123, Italy

## Abstract

One hundred years after the original formulation by Petrus J.W. Debije (aka Peter Debye), the Debye Scattering Equation (DSE) is still the most accurate expression to model the diffraction pattern from nanoparticle systems. A major limitation in the original form of the DSE is that it refers to a static domain, so that including thermal disorder usually requires rescaling the equation by a Debye-Waller thermal factor. The last is taken from the traditional diffraction theory developed in Reciprocal Space (RS), which is opposed to the atomistic paradigm of the DSE, usually referred to as Direct Space (DS) approach. Besides being a hybrid of DS and RS expressions, rescaling the DSE by the Debye-Waller factor is an approximation which completely misses the contribution of Temperature Diffuse Scattering (TDS). The present work proposes a solution to include thermal effects coherently with the atomistic approach of the DSE. A deeper insight into the vibrational dynamics of nanostructured materials can be obtained with few changes with respect to the standard formulation of the DSE, providing information on the correlated displacement of vibrating atoms.

Based on the orientational average of the intensity distribution, in 1915 Debye derived his equation for scattering^1^,


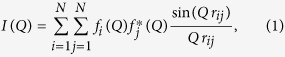


being *Q* = 4 *π* sin(Θ)/*λ* the wavevector transfer modulus (Θ is half the scattering angle and *λ* the radiation wavelength), *f* the atomic scattering factor and *r*_*ij*_ the magnitude of the distance between any two of the *N* atoms composing an atomistic aggregate.

Despite the apparent simplicity of equation 1, for many years the DSE was just a proof of concept, with few applications only (*e.g.* the pioneering electron diffraction study by Germer & White in^2^). As the summation runs over all *N* atoms, equation 1 requires calculation of *N*^2^ terms, which is computationally demanding if particles exceed a few tens of nanometers; moreover, the sample size and shape distributions must be properly represented, therefore implying a further increment in the number of terms to be computed.

However, in recent years the ever-increasing power of computers has alleviated computational problems, especially considering that equation 1 is ideally suited to parallel computing on manycore processing units^3^. At the same time, the modern trend in nanotechnology is to produce nanocrystalline powders of selected shapes, with a narrow dispersion of morphological and dimensional parameters^4^,^5^,^6^,^7^, therefore decreasing the degrees of freedom and increasing the level of detail of the required models. The effect of atomic vibrations has usually been included in a simplified way, multiplying equation 1 by the Debye-Waller (DW) factor^8^. For a monoelemental structure, as assumed in the following for simplicity and coherently with the experimental case study, the DW factor is written as 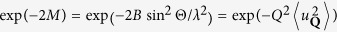, where 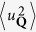 is the atomic Mean Squared Displacement (MSD) projected along **Q**, whereas the *B*-factor is defined as 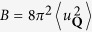.

The effect of the DW factor is to depress the diffracted (aka “Bragg”) intensity for the dynamic disorder brought in by the thermal displacement. While it is generally correct to provide for such an effect, two points should be considered. First, (i) the theory underlying the traditional DW factor is based on several simplifying assumptions, among which ignoring correlations between atomic displacements; furthermore, (ii) intensity removed from Bragg peaks should appear as a TDS signal. So far (i) has been largely ignored in DSE applications, whereas (ii) was only treated by approximate models in RS approach^9^.

In general terms, the instantaneous scattering amplitude from a small crystal is a sum of phase terms, weighted on the atomic scattering factors^8^,


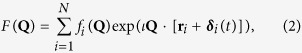


where the instantaneous atomic position, **r**_*i*_ + ***δ***_*i*_(*t*), includes a static component, **r**_*i*_, referred to an average position, plus a dynamic term, the time-dependent (thermal) displacement ***δ***_*i*_(*t*).

The squared modulus of the scattering amplitude is the scattered intensity, *I*(**Q**) = *F*(**Q**)*F*^ ∗^(**Q**), which has to be time-averaged to account for the interaction of photons (a fast probe, being their typical frequency considerably higher than that for atomic vibrations) with matter in a given experiment. Using compact notation, **r**_*ij*_ = **r**_*i*_ − **r**_*j*_ and ***δ***_*ij*_ = ***δ***_*i*_ − ***δ***_*j*_ (see Fig. 1),


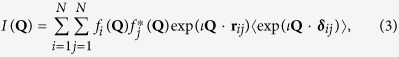


where the symbol 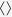 indicates the time average. If the vibration modes are independent, as it is true in the harmonic approximation, then the time-averaged phase term can be expanded in power series [ref. 10, p. 92–93], as


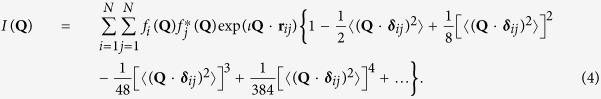


According to the above perturbation approach, the former term is the diffraction from the average (static) crystal whereas the following terms introduce thermal effects with increasing accuracy.

The intensity from an ideal powder of small crystals, *I*(**Q**), is given by the (spherical) orientational average of equation 4 over the angles *θ* and *ϕ* depicted in Fig. 1^1^,^8^. The crucial quantity is the square of the projection of ***δ***_*ij*_ over **Q**, which can be expressed as,


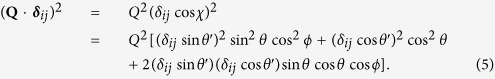


The scattered intensity, assuming spherical scattering factors and considering for the sake of discussion terms up to the second order in the series expansion, is therefore given by


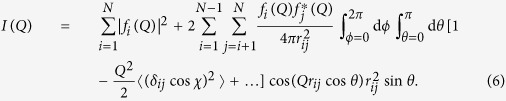


The solution of the integrals leads to the expression


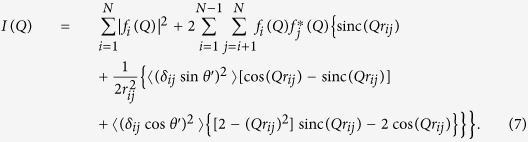


The time-averaged terms represent the square of the pair displacement projection respectively along the **r**_*ij*_ direction, 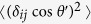, and perpendicular to it, 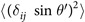, and therefore they can be related, respectively, to the longitudinal and transversal vibration modes for the given **r**_*ij*_.

Equation 7 can be written in a simpler form by considering that the time averages of amplitude (related to the temperature) and orientation of the displacement are independent, *i.e.* 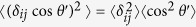 and 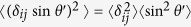. The assumption that atomic vibrations occur with the same probability in time along any direction (a condition which can be relaxed if necessary as reported in the Supplementary Information) leads to a further simplification, *i.e.* 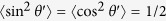.

The time average of 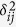 requires more consideration. Being the square modulus of the difference between two vectors, 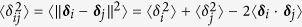. Within the discussed monoelemental case 
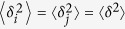
, whereas 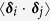 is the correlation between atomic displacements, expected to be significantly different from zero for close neighbors and in general for correlated atomic displacements. This suggests expressing 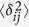 introducing the dimensionless parameter *k*_*ij*_,


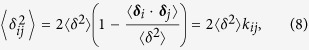


which can be calculated separately for different coordination shells. Denoting with the apex (*s*) values associated to the *s*-th shell *S*_*s*_, equation 7 can be expressed as


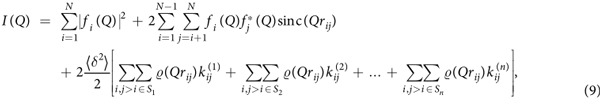


where 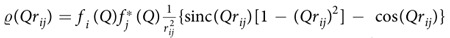 – expression including terms up to 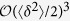 in the series expansion of equation 4 is expressed by equation S20.

The *k*_*ij*_ values account for the correlation between atomic vibrations of neighboring atoms, which depends on the vibration directions of the two neighbor atoms (equation 8). Correlation is maximum when 
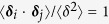
 (parallel displacements) or −1 (antiparallel displacements) and therefore *k*_*ij*_ = 0 or 2, whereas is null when the time-averaged scalar product is zero and *k*_*ij*_ = 1. In a general sense, values of *k*_*ij*_ between 0 and 1 correspond to acoustic vibrations (in-phase displacements of neighboring atoms) with numbers below unity limiting the depressing effect of thermal vibrations on Bragg peaks as atoms tend to move coherently therefore preserving the crystal order. On the other hand, *k*_*ij*_ values between 1 and 2 would be expected for optical vibrations, as in aggregates composed of different atomic species.

It is interesting to validate the approach empowered by equation 9 against an *in silico* case of study, the powder diffraction pattern of a palladium sphere (diameter 7.40 nm), obtained by taking the average of the outputs of equation 1 applied to a set of snapshots of a Molecular Dynamics (MD) trajectory at 300 K (see Supplementary Information), so to include the effect of atomic vibrations. To avoid dealing with a model for atomic displacements induced by the finiteness of the body^11^,^12^,^13^ and focus on thermal effects only, the input for the Thermal Debye Scattering Equation (TDSE, equation 9) was the aggregate obtained by averaging atomic positions over the same set of snapshots used to compute the diffraction pattern. Thermal effects were then modeled in terms of 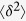 and *k*_*ij*_ parameters.

Figure 2a depicts the powder pattern from the simulated palladium nanosphere, together with the modelling by the TDSE obtained adjusting the value of *k*_*ij*_ parameters corresponding to the first twenty-five shells and 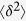. As explained in detail in the Supplementary Information, the modeling was performed by the StoRM code^14^, whose effectiveness and overall fit quality is demonstrated by a nearly zero and featureless residual reported in Fig. 2a. Most importantly, as illustrated in Fig. 2b, the refined 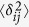 values closely match those computed from the MD trajectory therefore validating the proposed approach. As expected, the correlation degree decreases when increasing the pair distance and the *k*_*ij*_ values asymptotically tend to unity. It should be noticed that each *k*_*ij*_ refers to atoms lying along equivalent crystallographic directions (e.g. 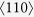 for the first fcc shell), so that it encodes information on the atomic distribution and anisotropy of the vibrations.

The experimental case is a powder of palladium nanocrystals. A Transmission Electron Microscopy (TEM) analysis (Fig. 3) on more than 1,000 items reveals that the nanoparticles are chiefly cubes, with rounded or truncated edges and corners^13^, whereas non-cubic objects are just few (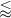
2%) smaller objects, which occupy an even smaller (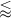
1%) volume fraction. The size distribution is little dispersed (s.d. ≈1.8 nm) around a mean edge of 15.1 nm. Based on this information, the TDSE was applied to a representative selection of truncated-nanocubes, using the StoRM code to match the experimental pattern collected at the 11-BM-B beamline at the Advanced Photon Source.

As reported in Fig. 4a, the TDSE properly matches the experimental pattern. Besides a low residual, the modelling accurately maps some expected features, like the interference fringes around the (200) Bragg peak^13^. These are “fingerprints” of the parallel cube faces, which are visible in a powder pattern from billions of palladium nanocubes because they are nearly identical in shape (Fig. 3), with little dispersion around the mean size.

The most recurrent particle is depicted in Fig. 4b together with the distribution of static displacement caused by the low coordination of surface atoms. As recently demonstrated, strain is highest on the surface but extends inside the nanocrystal with a complex trend which depends both on elastic anisotropy and on the aggregate size and shape^12^. The refined size distribution is superimposed on the TEM histogram distribution in Fig. 4c. The good match is a valuable confirmation of the validity of the proposed approach, but is also an expected result, as the starting configuration and the nanocrystal model were directly inspired by the TEM micrographs.

Information on the correlated thermal displacement is depicted in Fig. 4d, where the first 15 *k*_*ij*_ values refined by the StoRM code (red circle) are reported as a function of the shell number and interatomic distance. The same figure also shows MD values for the most representative truncated cube of the distribution in Fig. 4c (15.06 nm edge, blue circle), and a histogram of the number of items for each shell, 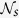. Values of *k*_*ij*_ markedly below unity demonstrate the strong correlation in the thermal motion of neighbor atoms, which extends to several shells before gradually approaching the uncorrelated condition (*k*_*ij*_ = 1).

Figure 4d also reports the continuous trend computed from a correlated-Debye (CD) model^15^. Based on rather simplified assumptions^15^,^16^, the CD model has just one adjustable parameter, the Debye temperature Θ_*D*_, which can be refined to give the best-match with the observed MSD data. For the discussed case of study, the best fit of 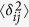 values gives Θ_*D*_ = 235 K, significantly lower than the literature value of 272(18)K for bulk palladium^17^. The difference is due to the small size of the nanoparticles: besides the effect of phonon confinement caused by the finite size, reduced surface coordination further contributes to increase the average MSD and, consequently, to reduce the average Debye temperature^11^.

The TDSE also provides a more accurate *B*-factor, with respect to that obtained from the DW factor, by averaging 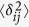 on the entire set of neighbor shells,


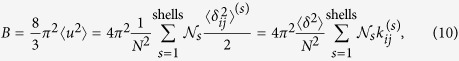


with the normalization resulting from 
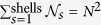
, the square of the total number of atoms. The *B*-factor for the powder of palladium nanocubes is 0.62 Å^2^, higher than the literature reference for the bulk phase (*B* = 0.45(6) Å^2 ^17) and comparable to literature values for nanocrystalline palladium^13^,^18^. Reasons for the increased value can be ascribed to phonon confinement and surface effects common to nanocrystals^11^,^19^,^20^,^21^,^22^. However, it is worth noting the literature values have been obtained from a traditional DW factor, whereas equation 10 and the TDSE provide a better average, made over the different coordination shells, correctly accounting for diffuse as well as Bragg scattering.

Differences in *k*_*ij*_ values between experimental results and MD simulations reported in Fig. 4d reveal the interaction of the particles with a complex surface environment (*e.g.* the capping agent protecting the particles), not considered in the simulation. Therefore, experimental values of the *k*_*ij*_ parameters, and particularly the deviation from the CD model, grant access to information on the atomic displacement correlation in nanostructured particles, related to the phonon dispersion^23^ and the interaction with the surrounding environment: this is definitely a step forward with respect to traditional powder diffraction methods, which hardly consider more than an average DW factor.

While powder diffraction is not usually credited for studying vibrational properties, traditionally measured by inelastic scattering techniques^10^,^24^, requiring single crystals, it could successfully reveal structural and dynamical features of nanostructured particles characterized by limited morphological dispersion. In the last few years, data collected up to high *Q* values have been employed to investigate correlated atomic displacements by means of the atomic Pair Distribution Function (PDF) approach^25^,^26^,^27^,^28^,^29^. In principle, this method gives information similar to that provided by the TDSE, showing details of the phonon dispersion for simple metal systems^23^. In fact, PDF analysis and DSE belong to the so-called Total Scattering methods, with the common root of the DS approach, based on interatomic distances. However, the PDF method appears more appropriate to investigate thermal properties of materials in the form of bulk-phase, well-crystallized powders^26^,^28^, for which the broadening of the PDF peaks is ascribed to thermal displacement and is properly modeled by a Gaussian profile^30^. Things can be quite different for nanocrystalline powders, where the PDF fades away for increasing distances, as an effect of the finite size, while peaks broaden for the cumulative effect of static and thermal features, with the anisotropic components disappearing in the representation offered by the PDF. On the contrary, the TDSE approach ideally pertains to nanoparticle systems and does not require high-*Q* data. Distinctive features of the microstructure, like those related to domain shape (*e.g.*, the interference fringes affecting the (200) peak in Fig. 4a) and anisotropy of atomic displacement, which modifies the line profile with specific dependence on the crystallographic direction, are directly observed in the powder pattern and can be properly accounted for if suitable physical models are implemented. Interestingly, information on the correlated atomic displacement (reflected by the *k*_*ij*_ parameters) affect the entire powder pattern, involving both diffuse and Bragg intensity, not only peak broadening. This fact is illustrated by Fig. 5a, where the static (equation 1) and thermal components of the pattern associated to the particle depicted in Fig. 4b are reported. The contribution of the first five shell in equation 9 is further highlighted in Fig. 5b, where each curve has to be weighted on the corresponding *k*_*ij*_ (Fig. 4c), therefore underlying that the curves associated to each term are distributed across the whole accessible range. This causes powder diffraction to be quite sensitive to even small changes in the *k*_*ij*_ values, providing stability to the method (a detailed analysis of residuals is reported in the Supplementary Information).

In conclusion, the presented extension to the Debye scattering equation (i) grants access to information on the correlated thermal displacement in nanostructured particles and (ii) concurs to enhance and strengthen the detail of information retrieved from scattering data, as demonstrated in Fig. 4. Static and thermal displacements can be rigorously investigated for systems of nanocrystals characterized by different sizes, shapes and surface interactions (*e.g.* capping agents). Even more interestingly, the effect of temperature on vibrational properties can be evaluated and the analysis of their modification as a consequence of the interaction with a specific environment (*e.g.* during oxidation/reduction or *in operando*) can be performed, therefore shedding new light on *in vivo* structural and dynamical features of nanostructured particles.

## Additional Information

**How to cite this article**: Scardi, P. and Gelisio, L. Vibrational Properties of Nanocrystals from the Debye Scattering Equation. *Sci. Rep.* **6**, 22221; doi: 10.1038/srep22221 (2016).

## Supplementary Material

Supplementary Information

## Figures and Tables

**Figure 1:**
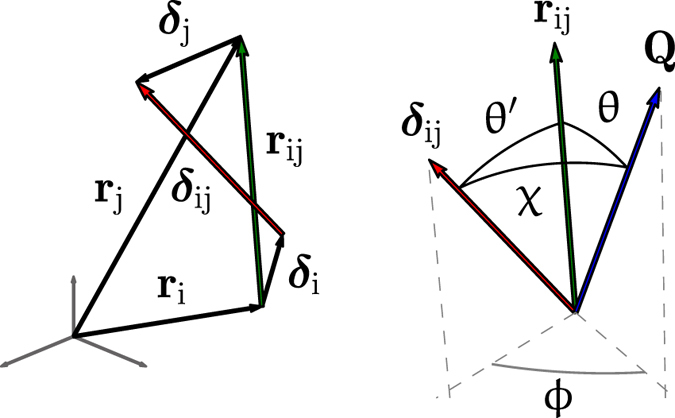
Geometrical relations among vectors ***δ***_*ij*_, **r**_*ij*_ (left), and **Q**, together with angles definitions (right).

**Figure 2:**
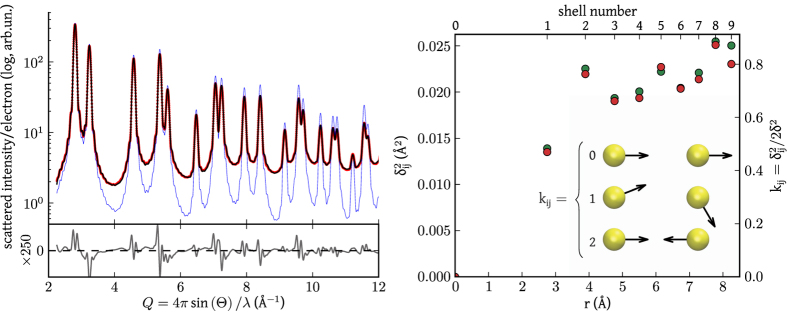
Atomic vibrations. Left, powder diffraction pattern computed from the MD simulation of a Pd nanosphere (red dots) together with the modeling by the TDSE (equation 9) fitting twenty-five *k*_*ij*_ parameters (the condition *k*_*ij*_ = 1 is assumed for all other shells) and 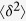 (black line). Their difference (residual, multiplied by 250 times) is reported below. The pattern from the corresponding “static” particles is also shown (blue line, 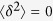 or equation 1). Right, 
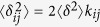
 values associated to the first shells computed from the MD trajectory (green) and from the TDSE fit (red). The geometrical meaning of *k*_*ij*_ parameters is also sketched.

**Figure 3:**
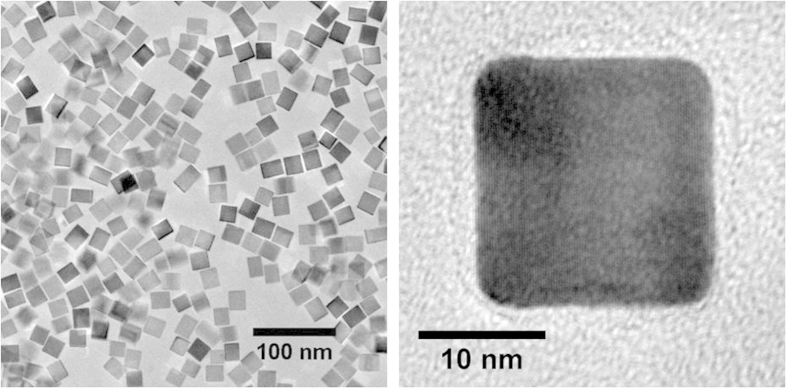
Pd nanoparticles composing the investigated sample. Non-cubic objects are infrequent, and even when observed they are small and nearly negligible in terms of volume fraction.

**Figure 4:**
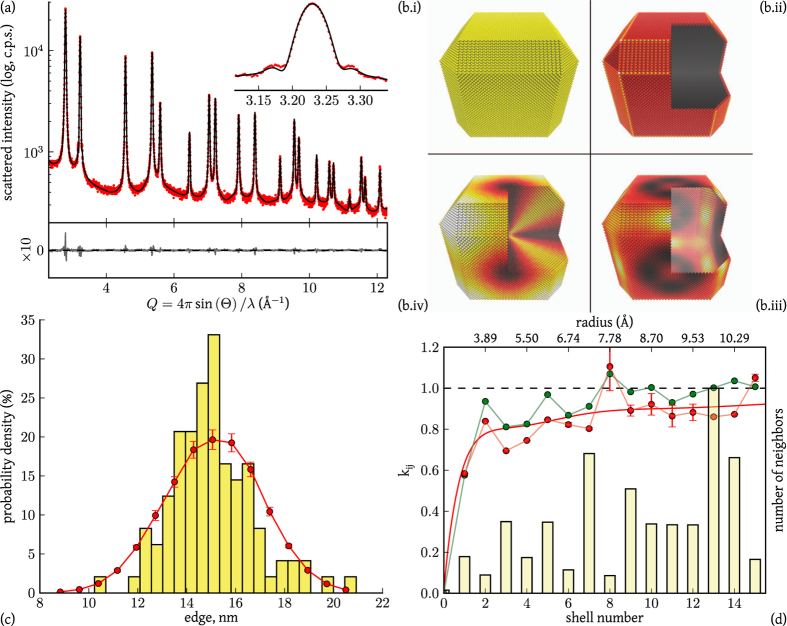
XRPD data modeling results. Experimental X-ray powder pattern (dot) is overlaid with the modeled pattern (line). The difference (residual, multiplied by ten times) is reported below, while the inset illustrates a detail of the (200) Bragg reflection (**a**). In (**b**), clockwise direction, (i) the most representative particle (15.06 nm) together with (ii) a qualitative view of the average atomistic strain (equation S13) obtained by fitting parameters *α*, *a*, *σ* in equation S9 to experimental data. Contribution of the “surface”-term containing *σ* (iii) and the one containing *a* (iv) are also presented (details in the Supplementary Information). Color scales are independent across representations and are implemented to highlight the complexity of the strain field. The cube edge histogram distribution obtained from TEM images (1,000 nanoparticles) is shown in (**c**), together with the result of the TDSE modeling (red circles). Picture (**d**) shows the number of items in each shell (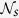, bar) and refined *k*_*ij*_ values as a function of the interatomic separation for experimental data (red) and MD (green), together with the Correlated Debye (CD, see text for details) trend for refined values (red line). Error bars in (**c**,**d**) are standard deviations over ten StoRM runs.

**Figure 5:**
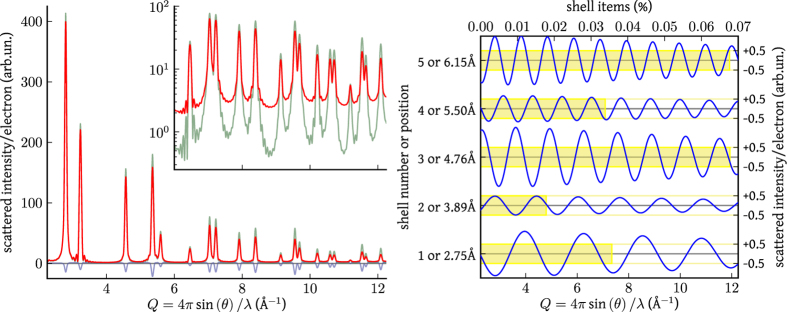
Thermal Debye scattering equation (red, equation 9) applied to the particle depicted in Fig. 4b and obtained by summing the static (green, equation 1) and thermal (blue) components (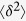 and *k*_*ij*_ values are taken from Fig. 4d) (a). Components of the Thermal Debye Scattering Equation (blue, curves are shifted for clarity) to be weighted by 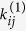 to 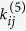. The wavelength of each function decreases when increasing the number of *k*_*ij*_ parameters whereas its amplitude is mostly proportional to the number of items in a given shell, depicted by a (yellow) bar (**b**).
