# Supplementary material for: Vibrational Properties of Nanocrystals from the Debye Scattering Equation
Source: Sci Rep. 2016 Feb 26;6:22221. doi: 10.1038/srep22221 (PMC4768180; doi:10.1038/srep22221)
Supplement: Supplementary Information [file srep22221-s1.pdf]

Vibrational properties of nanocrystals from the Debye Scattering Equation

P.Scardi and L.Gelisio
University of Trento

Methods and Supplementary Information

Synthesis of the particles and transmission electron microscopy characterization

Both the synthesis and the TEM characterization of Palladium nanoparticles were performed by B.T. Sneed, M.K. Sheehan and C.-K. Tsung at the Boston College. The procedure for the synthesis, adapted from [1], is reported in detail in [2].
A JEOL JEM2010F microscope operated at 200kV was employed to characterize the particles, drop-casted onto a carbon-coated copper grid.

High-resolution X-ray powder diffraction data collection

High-resolution XRPD data were collected at 11-BM-B [3, 4] at the Advanced Photon Source (Argonne National Laboratory). A Kapton® capillary (≈20-30mm length, 0.80mm diameter) was filled with a concentrated particle dispersion which was then allowed to dry for a few days. The capillary was installed on the beamline spinner (4,200rpm), to improve uniformity, and two measurements of one hour each were performed at room temperature (298K), counting 0.3s for each step. Collected data were binned so to further lower the signal-to-noise ratio. The (transmission) Debye-Scherrer geometry was implemented, using a detector system based on 12 independent Si(111) analyzers, and the wavelength of the impinging beam was 0.0413874nm (≈30keV).
Furthermore, to assess the sample absorption, the transmitted signal through the sample was collected after aligning the detector with the X-ray beam. The transmitted beam fraction was $I/I_0 = 0.974$ ($\mu R \approx 0.01$), low enough to make any correction for the absorption effect not necessary.

Atomistic simulations

The Large-scale Atomic/Molecular Massively Parallel Simulator (LAMMPS, [5]) code was employed to simulate the dynamics of 16,727 Palladium atoms arranged in a fcc (lattice parameter $a = 3.89$Å) sphere of 19 unit cells (7.40nm) diameter. Atomic interactions were ruled by the Embedded Atom Method (EAM, [6, 7], with interatomic potential from [8]) and the system was evolved in the micro-canonical (NVE) ensemble using 1fs timestep at 300.00 ± 0.13K.
The same procedure was reiterated to the most representative aggregate of the set employed to model scattering data, the particle depicted in figure M4b and 1b, composed of 197,587 atoms (the cube edge was 38 unit cells or 15.057nm and $T = 300.07 \pm 1.01$K).

Time-average scattering data The time-averaged pattern was computed by averaging the outputs of the DSE (equation M1) applied to 500 snapshots of the trajectory separated by 1ps. The (spherical) coherent scattering factor was

$$f_0(Q) = \sum_{i=1}^{4} a_i \exp\left(-b_i \frac{Q^2}{4\pi^2}\right) + c, \tag{1}$$

with $a_1 = 19.3319$, $b_1 = 0.698655$, $a_2 = 15.5017$, $b_2 = 7.98929$, $a_3 = 5.29537$, $b_3 = 25.2052$, $a_4 = 0.605844$, $b_4 = 76.8986$ and $c = 5.26593$ from [9].

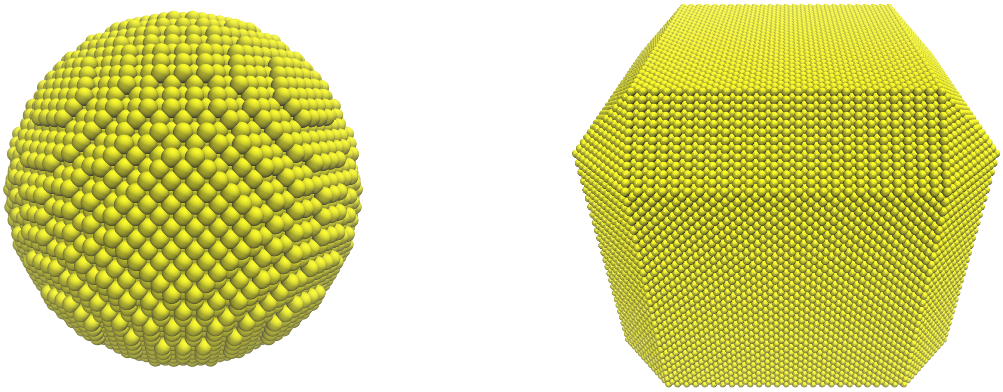

Figure 1: **Atomistic Palladium aggregate.** Representation of the 16,727 atoms Palladium sphere. Right, the most representative particle of the set employed to model experimental data, 15.057nm edge. Size and shape were inspired by TEM images.

Atomistic thermal quantities The position of atom i at a given instant t, $\mathbf{r}_i(t)$, can be broken down into a time-averaged component, $\mathbf{r}_i = \langle \mathbf{r}_i \rangle$, and a thermal displacement, $\boldsymbol{\delta}_i(t)$ (see figure M1a),

$$\mathbf{r}_i(t) = \mathbf{r}_i + \boldsymbol{\delta}_i(t). \tag{2}$$

Correlation of thermal displacements can be characterized both evaluating the projection of the displacement of i-th atom on that of atom j, $\boldsymbol{\delta}_i(t) \cdot \boldsymbol{\delta}_j(t)$, and the pair thermal displacement,

$$\boldsymbol{\delta}_{ij}(t) = \mathbf{r}_{ij}(t) - \mathbf{r}_{ij}, \tag{3}$$

with $\mathbf{r}_{ij}(t) = \mathbf{r}_i(t) - \mathbf{r}_j(t)$ and $\mathbf{r}_{ij} = \langle \mathbf{r}_{ij} \rangle = \langle \mathbf{r}_i \rangle - \langle \mathbf{r}_j \rangle$. The dimensionless coefficient

$$k_{ij} = 1 - \frac{\langle \boldsymbol{\delta}_i \cdot \boldsymbol{\delta}_j \rangle}{\langle u^2 \rangle} = \frac{\langle \boldsymbol{\delta}_{ij} \rangle}{2\langle u^2 \rangle} \tag{4}$$

is also defined, with $\langle u^2 \rangle$ being the average Mean Squared Displacement (MSD).
It must be noticed that being the above averages computed in a three-dimensional space whereas the quantity in equation M8 resides in a two-dimensional space, a conversion factor is needed to link atomistic and scattering quantities, *e.g.* for the MSD

$$\langle \delta^2 \rangle = \frac{2}{3} \langle u^2 \rangle. \tag{5}$$

The average number of atomic pairs separated by a distance r at time t is given by the Pair Distribution Function (PDF *aka* radial pair correlation function, see *e.g.* [10]),

$$g(r) = \frac{1}{\rho N} \sum_{n=1}^{N} \sum_{\substack{m=1 \\ m \neq n}}^{N} \delta\left(\|\mathbf{r}_{nm}(t)\| - r\right) \tag{6}$$

being $\delta(x)$ the Dirac-delta function [11], N the number of atoms composing the aggregate and ρ the numeral density. The PDF was applied to the same set of frames used to compute the time-averaged scattering pattern therefore obtaining a time-averaged pair distribution function. Coherently with the enforced harmonic approximation, to provide a different view and compare results of motion correlation from scattering data analysis, PDF peaks were fitted using Gaussian functions (see *e.g.* [12] and figure 2). Given the variance σ_{ij}^2, the relation

$$2\sigma_{ij}^2 = \frac{3}{2} \delta_{ij}^2 \tag{7}$$

allows to compare the different views.

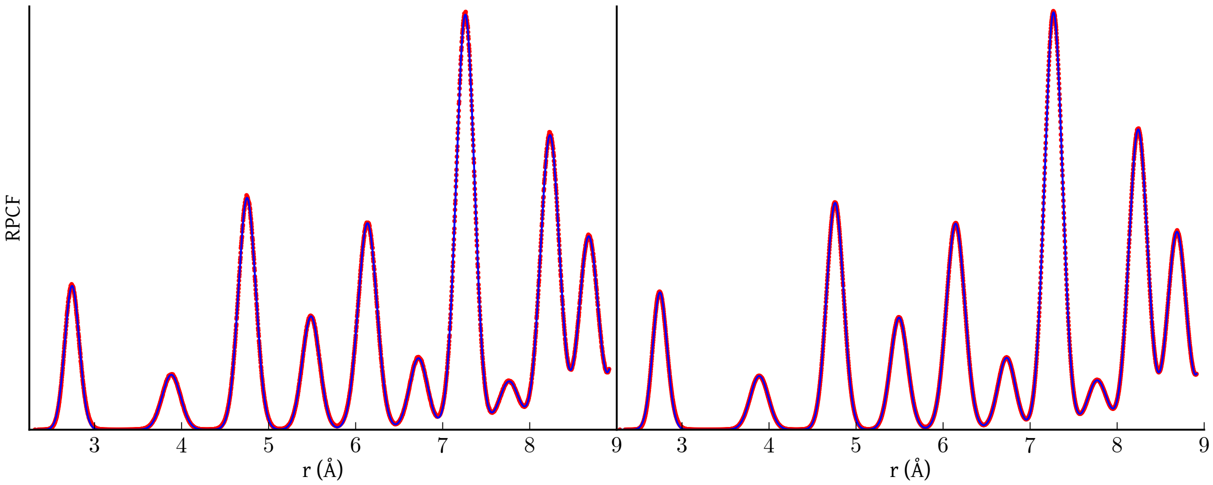

Figure 2: **Time-averaged pair distribution function** for the 16,727 atoms Palladium sphere (left) and for the most representative particle of the set employed to model experimental data, 15.057nm edge (right). In both cases, red dots represent the calculated function and blue lines are the Gaussian curves.

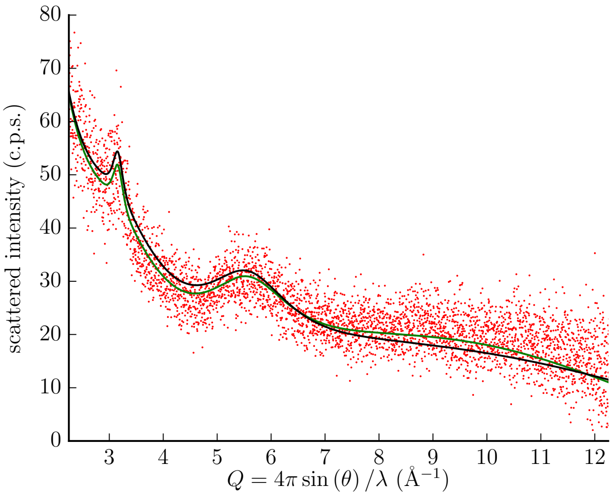

Figure 3: **Background modeling.** Scattering pattern of an empty Kapton® capillary (red dots), together with the optimized fitting function (black line). The green line represents the background function obtained after modeling the signal emanated from Palladium nanoparticles.

Scattering data modeling

Both simulated and experimental scattering data were modeled using the Stochastic Real-space Modeler (StoRM, [13]) code, implementing a *simulated annealing* algorithm [14–16]. The quantity to be minimized is the difference between intensity observed (I_o) and computed for a given model (I_m),

$$\chi^2 = \frac{1}{P-\nu}\sum_{p=1}^{P}\left(\frac{I_{m,p}-I_{o,p}}{\sigma_p}\right)^2, \tag{8}$$

being σ the standard uncertainty, P the number of pattern points and ν the varied parameters. Following, the most important aspects when dealing with experimental data are reported. Starting from a careful analysis of High-Resolution Transmission Electron Microscopy (HRTEM) pictures, sixteen (atomistic) particles were built according to crystallographic principles. The form factor associated to each point scatterer was the same described by equation 1.

Background modeling Air scattering and Kapton® contribution were accounted for by measuring an empty capillary. Data were fitted with a mixture of mathematical functions, namely seven pseudo-

Voigt curves and a fourth-order Chebyshev polynomial (definitions are in [13]).
This fit function was then perturbed with a third-order Chebyshev polynomial of the first kind during the modeling of data emanated from Palladium nanocubes and added to the output of the TDSE. The two curves are reported in figure 3, to highlight the background contribution.

Deformation of crystallographic particles The position of the i-th atom in the deformed configuration can be expressed as [13, 17, 18]

$$\mathbf{r}_{n,d}\left(\mathbf{r}_n, z; \alpha, a, \sigma\right) = \kappa\left(\hat{\mathbf{r}}; \alpha\right)\left[\frac{a - a_0}{a_0} + \sigma \ln\left(\frac{\mathcal{N}}{z}\right)\right]\mathbf{r}_n. \tag{9}$$

The fit parameter a is responsible for a constant offset of the deformation which insists on the particle, normalized for convenience to the bulk lattice parameter a_0, whereas σ modulates a term due to Pauling [19, 20], not null for undercoordinated atoms, *i.e.* atoms with less than $\mathcal{N} = 12$ (for the fcc system) nearest neighbors. The function κ produces a hkl-dependent behavior,

$$\kappa\left(\hat{\mathbf{r}}; \alpha\right) = (1 - \alpha) + \alpha\,\frac{s_{\hat{\mathbf{r}}}}{\overline{s}}, \tag{10}$$

being $s_{\hat{\mathbf{r}}}$ the compliance of the crystal projected on direction $\hat{\mathbf{r}}$, $\overline{s}$ its average value over all possible crystal directions and α a fit parameter. Considering cubic crystal symmetry, the reciprocal of the Young's modulus along $\hat{\mathbf{r}} = \{u_1, u_2, u_3\}$ can be expressed in terms of compliance matrix ($\mathcal{S}$) elements as (see *e.g.* [21]),

$$s_{\hat{\mathbf{r}}} = \frac{1}{E_{\hat{\mathbf{r}}}} = s_{11} - 2\left(s_{11} - s_{12} - \frac{s_{44}}{2}\right)\left(u_1^2u_2^2 + u_2^2u_3^2 + u_3^2u_1^2\right). \tag{11}$$

The average atomistic strain can then be evaluated by computing the difference of bond length (the Euclidean norm of $\mathbf{r}_{ij} = \mathbf{r}_i - \mathbf{r}_j$, i being a nearest neighbor of j) and averaging the quantity over the number of nearest neighbors,

$$b_n = \frac{1}{\mathcal{N}_n}\sum_{m=1}^{\mathcal{N}_n}\left\|\mathbf{r}_{nm}\right\|. \tag{12}$$

The average strain of atomic bonds of a given atom n as seen by its nearest neighbors can therefore be expressed as

$$\varepsilon_{b,n} = \frac{b_\mathrm{d}}{b_0} - 1. \tag{13}$$

Varied parameters To summarize, 5 parameters were fitted to account for the background (1 for scaling the fit function and 4 for a third-order Chebyshev polynomial), 16 to define each particle fraction and 5 for the deformation model. Indeed, both values of a and σ were mapped on different sizes through a Young-Laplace-like law [22, 23],

$$\phi = \phi_0 + \frac{\phi_s}{e} \tag{14}$$

being e the edge of the cube and ϕ either a or σ.
The number of parameters for vibrational properties ranged from 2 ($\langle\delta^2\rangle$ and Θ for the Debye correlated model) to the chosen number of k_{ij} values to be refined plus one ($\langle\delta^2\rangle$).

Sensitivity to k_{ij} parameters For a given set of varied parameters, data modeling was reiterated ten times with different seeds for the (ranlux [24]) pseudo-random number generator, implemented within the GNU Scientific Library [25]. To assess the effect of the number of fittable k_{ij} parameters on residuals, *i.e.* the difference between intensity observed and computed for a given model, equation 8 was evaluated for each configuration. Figure 4a illustrates the evolution of χ^2 when progressively increasing the number of k_{ij} parameters different from unity together with the fraction of neighbors

in a given shell, which reveals the sensitivity to each parameter, as demonstrated from a different point of view in figure M5.
To conclude the analysis, figure 4b illustrates the output of equation M9 applied to the particle depicted in figure 1, both implementing the uncorrelated model ($k_{ij} = 1$ for each shell) and using fifteen k_{ij} parameters from experimental data modeling.

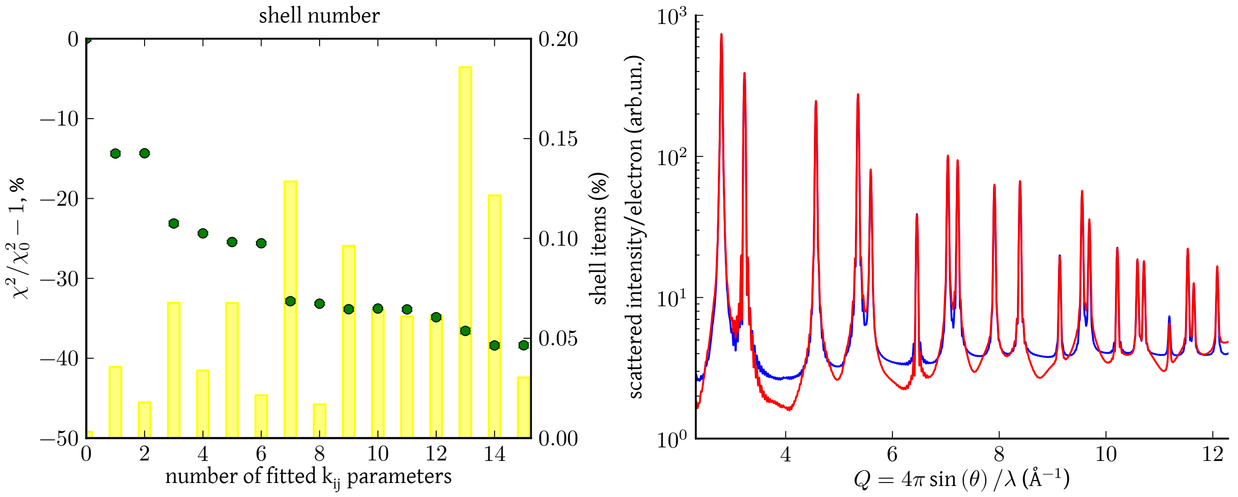

Figure 4: ***k_{ij}* parameters effect on χ^2.** Left, reduction trend of χ^2 when increasing the number of k_{ij} parameters, from zero (corresponding to the uncorrelated model since the relation $k_{ij} = 1$ holds for all parameters) to fifteen. Yellow bars depict the population of a given coordination shell (the most representative particle has been analyzed) and justify the relative weight of the pattern component associated to a given k_{ij} (see also figure M5b). Right, simulation of the scattering pattern emanated from the particle sketched in figure 1 assuming uncorrelated motion ($k_{ij} = 1$ for each shell) and with fifteen k_{ij} parameters from experimental data modeling.

Statistical analysis For the complete set of varied parameter, a typical χ^2 curve is reported as a function of the annealing temperature in figure 5a. Collecting only combinations of parameters giving a χ^2 *reasonably close* to the "best value", in this case values in $\left[\min\{\chi^2\}, 1.05\min\{\chi^2\}\right]$, *i.e.* inside the red region depicted in figure 5a, an unbiased variance-covariance matrix can be drawn by computing each coefficient as

$$C_{nm} = \sum_{i=1}^{N} \frac{(x_{ni} - \overline{x}_n)(x_{mi} - \overline{x}_m)}{N-1}. \tag{15}$$

The diagonal of the variance-covariance matrix defines the variance of a given entry n, reported in figure 5b for a selected set of parameters as coefficient of variation

$$\mathrm{CV} = \frac{\sigma}{\mu} = \frac{C_{nn}}{\mu}. \tag{16}$$

The Pearson product-moment correlation coefficients [26],

$$R_{nm} = \frac{C_{nm}}{\sqrt{C_{nn}\,C_{mm}}} \tag{17}$$

for a typical case are also reported in figure 6, limiting the analysis to the parameters describing the background (ι and a_0 to a_3), the particle fraction (ϕ_1 to ϕ_{16}) and atomic vibrations ($\langle\delta^2\rangle$ and $k_{ij}^{(1)}$ to $k_{ij}^{(15)}$). Interestingly, while background and particle fraction terms exhibit strong correlation, vibrational coefficients (especially $\langle\delta^2\rangle$ and k_{ij} values for low index neighbor shells) are rather uncorrelated from every parameter.

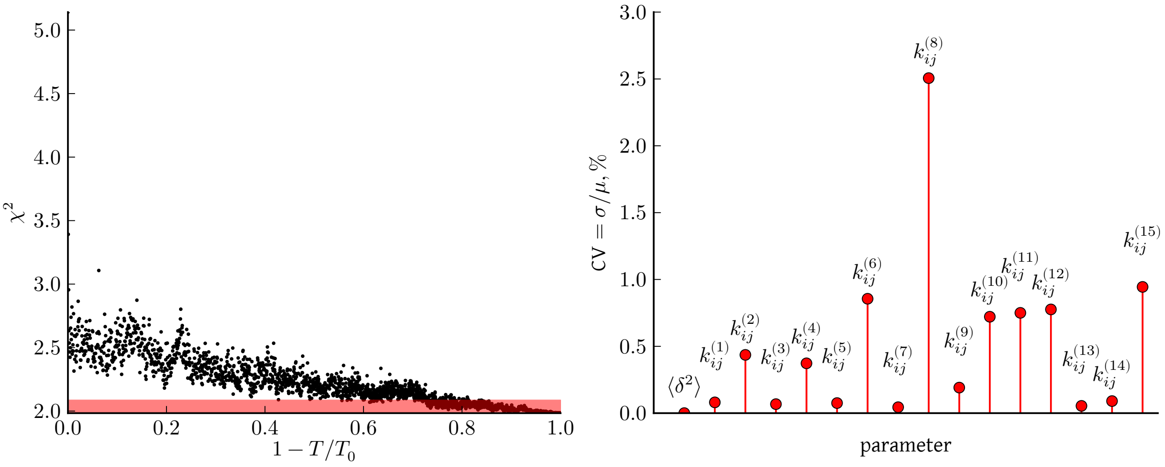

Figure 5: **χ^2 evolution as a function of temperature.** Left, reduction trend of χ^2 when decreasing the annealing temperature. Red region indicates values collected to compute the variance-covariance matrix (equation 15). Right, coefficient of variation (CV, equation 16) for selected parameters.

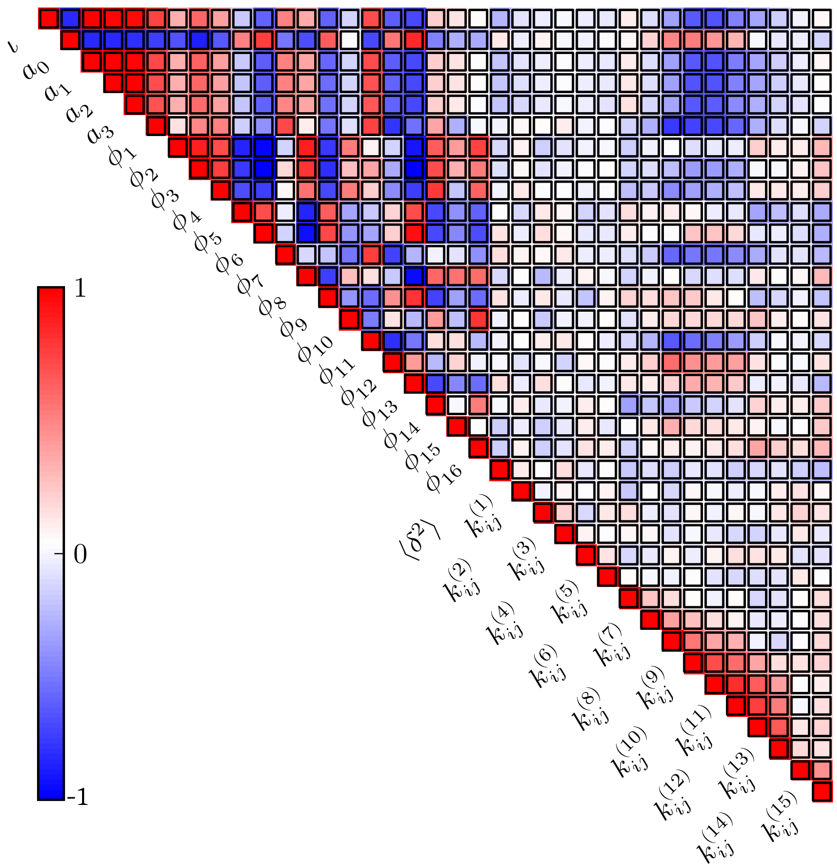

Figure 6: **Correlation of parameters.** Pearson product-moment correlation matrix for a selected set of parameters. The value of each coefficient is expressed by a red-white-blue color scale, being red equal to +1, white equal to 0 (uncorrelated values) and blue equal to −1.

Thermal Debye Scattering Equation

Longitudinal and transversal vibration modes Equation M9 can be generalized to allow for independent transversal and longitudinal (with respect to the bond axis) vibrations by relieving the condition $\langle \sin^2\theta' \rangle = \langle \cos^2\theta' \rangle = 1/2$. Therefore, k_{ij} parameters for longitudinal ($k_{ij}^{(L)}$) and transversal modes ($k_{ij}^{(T)}$) can be introduced in equation M7, leading to

$$\begin{aligned} I(Q) = &\sum_{i=1}^{N} |f_i(Q)|^2 + 2\sum_{i=1}^{N-1}\sum_{j=i+1}^{N} f_i(Q) f_j^*(Q)\,\mathrm{sinc}(Qr_{ij}) \\ &+ 2\frac{\langle\delta^2\rangle}{2}\left\{\sum\sum_{i,j>i\,\in\,S_1}\left[\varrho^{(L)}(Qr_{ij})\,k_{ij}^{(L1)} + \varrho^{(T)}(Qr_{ij})\,k_{ij}^{(T1)}\right] + \right. \\ &\sum\sum_{i,j>i\,\in\,S_2}\left[\varrho^{(L)}(Qr_{ij})\,k_{ij}^{(L2)} + \varrho^{(T)}(Qr_{ij})\,k_{ij}^{(T2)}\right] + \ldots + \\ &\left.\sum\sum_{i,j>i\,\in\,S_n}\left[\varrho^{(L)}(Qr_{ij})\,k_{ij}^{(Ln)} + \varrho^{(T)}(Qr_{ij})\,k_{ij}^{(Tn)}\right]\right\}, \end{aligned} \tag{18}$$

where,

$$\varrho^{(L)}(Qr_{ij}) = f_i(Q) f_j^*(Q)\frac{1}{r_{ij}^2}\left\{\cos(Qr_{ij}) - \mathrm{sinc}(Qr_{ij})\right\} \tag{19a}$$

$$\varrho^{(T)}(Qr_{ij}) = f_i(Q) f_j^*(Q)\frac{1}{r_{ij}^2}\left\{\left[2 - (Qr_{ij})^2\right]\mathrm{sinc}(Qr_{ij}) - 2\cos(Qr_{ij})\right\}. \tag{19b}$$

Series expansion of equation M4 with terms up to $\mathcal{O}\left(\langle\delta^2\rangle/2\right)^3$ Equation M9 can be easily made more accurate by adding higher order terms of the series expansion of equation M4, leading to

$$\begin{aligned} I(Q) = &\sum_{i=1}^{N} |f_i(Q)|^2 + 2\sum_{i=1}^{N-1}\sum_{j=i+1}^{N} f_i(Q) f_j^*(Q)\,\mathrm{sinc}(Qr_{ij}) + 2\sum\sum_{i,j>i\,\in\,S_1}\sum_{k=1}^{3}\frac{\langle\delta^2\rangle^k}{2^k}\frac{\varrho_k(Qr_{ij})}{r_{ij}^{2k}}k_{ij}^{(1)k} \\ &+ 2\sum\sum_{i,j>i\,\in\,S_2}\sum_{k=1}^{3}\frac{\langle\delta^2\rangle^k}{2^k}\frac{\varrho_k(Qr_{ij})}{r_{ij}^{2k}}k_{ij}^{(2)k} + \ldots + 2\sum\sum_{i,j>i\,\in\,S_n}\sum_{k=1}^{3}\frac{\langle\delta^2\rangle^k}{2^k}\frac{\varrho_k(Qr_{ij})}{r_{ij}^{2k}}k_{ij}^{(n)k}, \end{aligned} \tag{20}$$

being

$$\varrho_1(Qr_{ij}) = f_i(Q) f_j^*(Q)\left\{\left[1 - (Qr_{ij})^2\right]\mathrm{sinc}(Qr_{ij}) - \cos(Qr_{ij})\right\} \tag{21a}$$

$$\begin{aligned} \varrho_2(Qr_{ij}) = f_i(Q) f_j^*(Q)\frac{1}{2}\Big\{ &\left[-39 + 15(Qr_{ij})^2 + (Qr_{ij})^4\right]\mathrm{sinc}(Qr_{ij}) - \\ &\left[-39 + 2(Qr_{ij})^2\right]\cos(Qr_{ij})\Big\} \end{aligned} \tag{21b}$$

$$\begin{aligned} \varrho_3(Qr_{ij}) = f_i(Q) f_j^*(Q)\frac{1}{2}\Big\{ &\left[-285 + 165(Qr_{ij})^2 - 20(Qr_{ij})^4 - \frac{1}{3}(Qr_{ij})^6\right]\mathrm{sinc}(Qr_{ij}) - \\ &\left[-285 + 70(Qr_{ij})^2 - 3(Qr_{ij})^4\right]\cos(Qr_{ij})\Big\}. \end{aligned} \tag{21c}$$

The above expressions, including terms up to $\mathcal{O}\left(\langle\delta^2\rangle/2\right)^3$, are sufficiently accurate for the proposed case of study. However, adding further terms to the expansion of equation M4 is relatively straightforward, although it involves an increasing algebraic complexity.

Additional material

To highlight the effect of atomic vibrations on the powder diffraction pattern emanated from the most representative particle of the set employed to model experimental data, animation 1 illustrates the effect of k_{ij} values associated to the first three neighbor shells. Additionally, the line corresponding to uncorrelated motion (all the k_{ij} parameters equal to unity, green line) and the pattern corresponding to a static object ($\langle\delta^2\rangle = 0\text{Å}^2$, blue line), implying the TDSE to reduce to the DSE, are also reported.

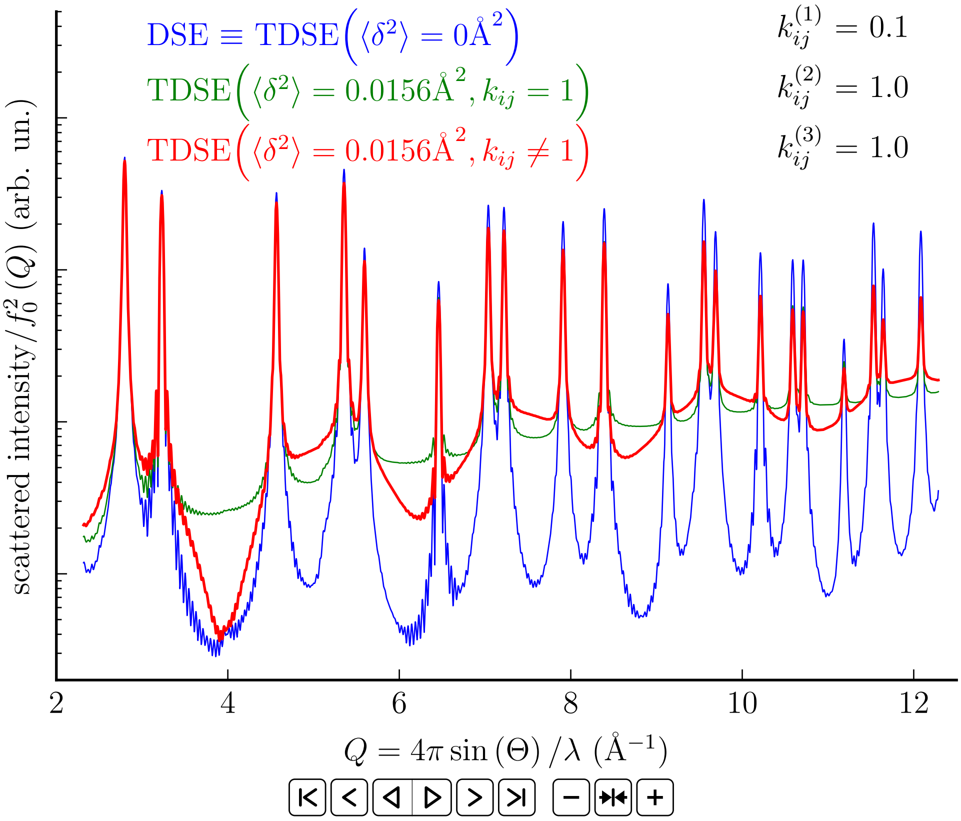

Animation 1: **Correlation of atomic vibrations: Effect of $k_{ij}^{(1)}$ to $k_{ij}^{(3)}$ parameters on the powder diffraction pattern** for the most representative particle of the set employed to model experimental data. If atoms are fixed, $\langle\delta^2\rangle = 0\text{Å}^2$ and the TDSE (equation M9) reduces to the DSE (blue line). The line profile associated to atoms vibrating independently (uncorrelated motion) and according to a Gaussian distribution is depicted by the green line ($\langle\delta^2\rangle = 0.0156\text{Å}^2$ and every k_{ij} equals unity). Given the same $\langle\delta^2\rangle$ of the previous case, the red line simulates different combinations of $k_{ij}^{(1)}$, $k_{ij}^{(2)}$ and $k_{ij}^{(3)}$.

References

1. Jin, M. *et al.* Synthesis of Pd nanocrystals enclosed by {100} facets and with sizes <10 nm for application in CO oxidation. *Nano Res.* **4**, 83–91 (2011).
2. Scardi, P. *et al.* Anisotropic atom displacement in Pd nanocubes resolved by molecular dynamics simulations supported by x-ray diffraction imaging. *Phys. Rev. B* **91**, 155414–155421 (2015).
3. 11BM at the Advanced Photon Source. *http://11bm.xray.aps.anl.gov/*
4. Wang, J. *et al.* A dedicated powder diffraction beamline at the Advanced Photon Source: Commissioning and early operational results. *Rev. Sci. Instrum.* **79**, 085105–085111 (2008).
5. Plimpton, S. Fast Parallel Algorithms for Short-Range Molecular Dynamics. *J. Comput. Phys.* **117**, 1–19 (1995).
6. Daw, M.S. & Baskes, M.I. Semiempirical, Quantum Mechanical Calculation of Hydrogen Embrittlement in Metals. *Phys. Rev. Lett.* **50**, 1285–1288 (1983).
7. Daw, M.S. & Baskes, M.I. Embedded-atom method: Derivation and application to impurities, surfaces, and other defects in metals. *Phys. Rev. B* **29**, 6443–6453 (1984).
8. Sheng, H.W., Kramer, M.J., Cadien, A., Fujita, T. & Chen, M.W. Highly optimized embedded-atom-method potentials for fourteen fcc metals. *Phys. Rev. B* **83**, 134118–134137 (2011).
9. Brown, P., Fox, A., Maslen, E., O'Keefe, M. & Willis, B. In *International Tables for Crystallography Volume C: Mathematical, physical and chemical tables* (ed Prince, E.) 554–595 (Springer Netherlands, 2004). doi:10.1107/97809553602060000600.
10. Allen, M.P. & Tildesley, D.J. *Computer Simulation of Liquids* (Clarendon Press, 1989).
11. Dirac, P.A.M. *The Principles of Quantum Mechanics. 4th ed.* (Oxford University Press, 1958).
12. Toby, B.H. & Egami, T. Accuracy of pair distribution function analysis applied to crystalline and non-crystalline materials. *Acta Crystallogr. Sect. A* **48**, 336–346 (1992).
13. Gelisio, L. *Structure and properties of nanostructured materials from atomistic modeling and advanced diffraction methods.* PhD thesis (2014).
14. Kirkpatrick, S., Gelatt, C. & Vecchi, M. Optimization by Simulated Annealing. *Science* **220**, 671–680 (1983).
15. Černý, V. Thermodynamical approach to the traveling salesman problem: An efficient simulation algorithm. *J. Optimiz. Theory App.* **45**, 41–51 (1985).
16. Corana, A., Marchesi, M., Martini, C. & Ridella, S. Minimizing Multimodal Functions of Continuous Variables with the 'Simulated Annealing' Algorithm. *ACM Trans. Math. Softw.* **13**, 262–280 (1987).
17. Gelisio, L., Beyerlein, K.R. & Scardi, P. Atomistic modeling of lattice relaxation in metallic nanocrystals. *Thin Solid Films* **530**, 35–39 (2013).
18. Gelisio, L. & Scardi, P. On the Modeling of the Diffraction Pattern from Metal Nanocrystals. *Metall. Mater. Trans. A* **45**, 1–10 (2014).
19. Pauling, L. Atomic Radii and Interatomic Distances in Metals. *J. Am. Chem. Soc.* **69**, 542–553 (1947).
20. Huang, W.J. *et al.* Coordination-dependent surface atomic contraction in nanocrystals revealed by coherent diffraction. *Nat. Mater.* **7**, 308–313 (2008).
21. Nye, J.F. *Physical Properties of Crystals: Their Representation by Tensors and Matrices* (Oxford University Press, 1985).
22. Young, T. An Essay on the Cohesion of Fluids. *Philos. Trans. Roy. Soc. London* **95**, 65–87 (1805).
23. Laplace, Marquis de, P.-S. *Traité de Mécanique Céleste* (Courcier, 1805).

24. Lüscher, M. A portable high-quality random number generator for lattice field theory simulations. *Comput. Phys. Commun.* **79**, 100–110 (1994).

25. Galassi, M. *et al. GNU Scientific Library Reference Manual. 3rd ed.* Network Theory Limited (2009).

26. Pearson, K. Mathematical Contributions to the Theory of Evolution. III. Regression, Heredity, and Panmixia. *Philosophical Transactions of the Royal Society of London A: Mathematical, Physical and Engineering Sciences* **187**, 253–318 (1896).

27. Hunter, J.D. Matplotlib: A 2D graphics environment. *Computing In Science & Engineering* **9**, 90–95 (2007).

28. Humphrey, W., Dalke, A. & Schulten, K. VMD – Visual Molecular Dynamics. *Journal of Molecular Graphics* **14**, 33–38 (1996).

Figures have been produced using Matplotlib [27] and VMD [28].
Only Open-source software has been used.
